# Human papillomavirus vaccination: if the vaccine is important and available, why not use it?

**DOI:** 10.1590/1806-9282.20240865

**Published:** 2025-05-02

**Authors:** Georgiana Sousa Freire, Carolina Lavacchini Ramunno Amaral, Juliana Jorge Romano, Camila Bussamra Aulicino, Cláudia Vaz de Melo Sette, Juliana Vieira Biason Bonometto, Jean Henri Maselli-Schoueri, Luiz Vinicius de Alcantara Sousa, Auro Del Giglio, Daniel de Iracema Gomes Cubero

**Affiliations:** 1 Centro Universitário Faculdade de Medicina do ABC – Santo André (SP), Brazil.; 2 Centro Universitário Faculdade de Medicina do ABC, Department of Oncology – Santo André (SP), Brazil.

**Keywords:** Immunization schedule, Papillomavirus vaccine, Papillomavirus infection, Public health, Brazil

## Abstract

**BACKGROUND:**

Human papillomavirus (HPV) is the most common virus of the reproductive tract, is linked to cervical cancer, and can be prevented by vaccination, which is most effective if the vaccine is administered before sexual activity begins.

**METHODS:**

This descriptive, cross-sectional, and qualitative study was based on a survey containing 15 questions delivered to schools in three cities in the ABC region. Two schools from high-income neighborhoods and two from low-income neighborhoods were selected in each city based on real estate values. Data were expressed in absolute numbers and percentages and interpreted by descriptive analysis. The statistical tests of association were performed.

**RESULTS:**

Twelve schools were invited and nine agreed to participate. Of the 4,503 questionnaires delivered, 1,921 were completed by parents and guardians. The vaccination rate was 56.05% in private schools and 66.58% in public schools. Private vs. public school was not an independent factor for vaccination, but residing in a low-income neighborhood and city was a determinant factor. Approximately 40% of the parents/guardians reported not having their children vaccinated, primarily due to concerns about adverse effects.

**CONCLUSION:**

Despite being freely available and proven effective, the human papillomavirus vaccine remains underutilized. The reasons exposed in this paper may be useful in strategies to enhance vaccination coverage.

**Trial registration:**

This study was approved by the research ethics committee under the number 2.143.196.

## INTRODUCTION

Human papillomavirus (HPV) is the most common reproductive tract virus and can be acquired by sexually active women and men^1^. It is the main cause of almost all cervical cancers and is responsible for a significant fraction of other genital and oropharyngeal cancers^2^. With approximately 570,000 new cases per year worldwide, cervical cancer is the fourth most common type of cancer among women worldwide^3,4^. It accounts for 311,000 deaths per year and is among the most frequent causes of cancer deaths in women^5,6^.

The most effective prevention strategy is the HPV vaccine. Currently, there are three vaccines available: quadrivalent (Gardasil^®^), bivalent (Cervarix^®^), and ninevalent vaccine, which is the only one not yet available in Brazil^7^. According to the World Health Organization (WHO), both the available vaccines are safe and may reach efficacy levels of 90–100% if administered before exposure to the virus^8–10^.

In this sense, the Brazilian Ministry of Health initiated free Gardasil^®^ vaccinations for girls aged 9-13 years through the Unified Health System in 2014. Subsequently, boys aged 11-15 years were included. However, despite being free, coverage with one dose decreased by 23% from 2014 to 2015, making it necessary to understand the social and structural barriers that caused such a decline^11,12^.

Therefore, this study aimed to explore the reasons why children are not being vaccinated and to aid the development of new approaches that encourage better adherence to immunization campaigns.

## METHODS

This descriptive, cross-sectional, and qualitative study received prior approval from the Research Ethics Committee under the number 2.143.196. All participants were informed about the objectives of the study and the use of its data.

### Sample selection

In this study, schools were chosen for two reasons: They serve as a gathering place for children of a specific age group and facilitate communication with parents and guardians and they allow for the evaluation of income's impact on public health knowledge.

For each city in the ABC Paulista (São Caetano do Sul, Santo André, and São Bernardo do Campo), two schools from a high-income neighborhood and two from a low-income neighborhood were selected based on the average value of the real estate in each region. Thus, the study involved a total of 12 schools (six public and six private) across six different neighborhoods (three high-income and three low-income).

### Data collection

We utilized a survey containing 15 questions about HPV to analyze the knowledge of the parents and guardians of children in the age group targeted by the public vaccination policy. The study was conducted in three stages:

1. Visits to selected schools to issue invitations to the board of directors to participate in this study.
2. Delivery of questionnaires to students in the fourth through ninth grades (age range: 9-14 years). The questionnaires were sent home via school diaries for guardians to complete and then were returned to teachers.
3. Collection of the completed questionnaires.

### Statistical analysis

Data collected from the questionnaires were tabulated, expressed in absolute numbers and percentages, and interpreted using a descriptive analysis. Differences between high- and low-income neighborhoods and between public and private schools were compared. A multivariate analysis was performed to evaluate the association between qualitative variables explored in the questionnaires.

## RESULTS

Of the 12 pre-selected schools, nine agreed to participate in the study¾six in high-income neighborhoods and three in low-income neighborhoods. Five schools were private and four were public. Of 4,503 questionnaires delivered, only 1,921 were completed.

### Profile of parents and/or guardians

The questionnaires were completed mainly by mothers. Regarding educational level, 50% of the respondents had completed higher education and 40% had high school diplomas. Approximately 64.7% had access to the private healthcare system (Table 1). The average age of the respondents was 41.9±7.3 years, and the families had up to four children, aged 6.2±5.3 to 12.2±4.2 years.

**Table 1:** Profile of the families participating in the study and human papillomavirus transmission according to them | *the (%) that equals less than 100 is due to "not informed" answers.

Variable			Frequency	Percentage (%)
Who answers				
	Mother		1.328	79.8
	Father		264	15.8
	Grandmother		51	3
	Grandfather		3	0.18
	Sister		3	0.18
Gender				
	Female		1.436	86.2
	Male		221	13.2
Education				
	Elementary education		161	9.6
	Secondary education		528	31.7
	Higher education		841	50.5
	Master's degree		93	5.5
	Doctorate		14	0.84
	Graduate degree		1	0.06
Medical care				
	Agreement		883	53
	Public health		428	27.3
	Private		134	8
	Agreement and public health		21	1.2
	Agreement and private		49	2.9
	Private and public health		7	0.4
	Private, public health, and health insurance		8	0.4
**HPV transmission according to participants**				
			**Frequency**	**Percentage (%)**
Way of transmission				
	Towel			
		Yes	239	14.4
		No	1,421	85.6
Sexual				
	Yes		1,618	97.6
	No		39	2.3
Saliva				
	Yes		232	14
	No		1,419	85.9
Insect				
	Yes		24	1
	No		1,626	99
Syringe				
	Yes		336	20.3
	No		1,315	79.6
Closed environments				
	Yes		8	0.4
	No		1,641	99.4

HPV: human papillomavirus.

### Knowledge about human papillomavirus

Almost everyone reported having some knowledge about HPV, with television as the most common source of information (74.1%). Majority of the respondents (68.5%) indicated that their information came from a doctor or other health professionals. When asked about which diseases HPV was related to, 87.7% of the participants said sexually transmitted diseases (STDs) and 62.6% said cancer.

### Vaccine against human papillomavirus

The vaccine availability in public and private networks was known by most participants: They knew the ideal age range for vaccination, its appropriate timing, and the guidelines provided by the Ministry of Health (Table 3). However, approximately 40% of the respondents reported not having vaccinated their children, primarily due to concerns about perceived adverse events (Table 2).

**Table 2:** Knowledge about the availability and indication for the human papillomavirus vaccine.

Question		Frequency	Percentage (%)
Related to sexual activity			
	Yes	1,511	92.6
	No	64	3.9
	I don't know	55	3.3
Is there a vaccine for women?			
	Yes	1,565	94.9
	No	15	0.9
	I don't know	69	4.1
Is there a vaccine for men?			
	Yes	1,246	76
	No	102	6.2
	I don't know	288	17.5
Availability			
	Private	36	2.2
	Public health	204	12.5
	Both	1,381	84.9
Are children vaccinated?			
	Yes	987	60
	No	623	37.9
	I don't know	33	2
Is there a fear of vaccination?			
	Yes	194	11.9
	No	1,436	88
Is there an ideal age range for vaccination?			
	Yes	1,423	86.9
	No	55	3.3
	I don't know	158	9.6
What is the age recommended by the MS*?			
	Newborns up to 1 year	13	0.8
	2-7 years	8	0.5
	9-13 years	1,587	97.1
	After 18 years	25	1.5
Do vaccines have to be made before the first sexual intercourse?			
	Yes	1,004	61.4
	No	314	19.2
	I don't know	313	19.1
Is there a benefit in receiving the vaccine after the recommended age?			
	Yes	1,271	77.5
	No	85	5.2
	I don't know	278	17

* MS = Ministério da Saúde.

**Table 3:** Vaccination coverage descriptives and multivariate analysis by logistic regression.

Variable		No		Yes		
		n	%	n	%	
Fear of vaccination?		1,669	88.54%	216	11.46%	
		**Vaccinated**		**Non-vaccinated**		
		**n**	**%**	**n**	**%**	
Total		1,147	60.37%	753	39.63%	
School						
	Public	518	66.58%	260	33.42%	p<0.001 (χ^2^)
	Private	629	56.05%	493	43.94%	
Neighborhood						
	Low income	714	69.25%	317	30.75%	p<0.001 (χ^2^)
	High income	433	49.83%	436	50.17%	
City						
	SCS	50	57.47%	37	42.53%	p<0.001 (χ^2^)
	SA	662	54.26%	558	45.74%	
	SBC	435	73.36%	158	26.64%	
**Multivariate analysis**						
**Vaccinated children**			**Odds ratio**	**Std. error**	**z**	**p>[z]**
School (public vs. private)			1.05	0.12	0.43	0.665
Neighborhood (low vs. high income)			1.85	0.25	4.59	<0.001
City (SCS, SA, and SBC)			1.36	0.15	2.81	0.005

SCS: São Caetano do Sul, SA: Santo André, SBC: São Bernardo do Campo.

### Vaccination coverage

Most of the guardians were not afraid to vaccinate their children, but only 60.37% of children were vaccinated. Public schools had the highest percentage of vaccinated students at 66.58%, compared to 56.05% in private schools. A multivariate analysis by logistic regression indicated that the type of school (private vs. public) was not an independent factor for vaccination, but residing in a low-income neighborhood was a significant determinant (Table 3).

## DISCUSSION

In the present study, we found that, contrary to what was expected, there was low adherence to a survey that could contribute to public health. There was also resistance from schools to deliver the questionnaires for logistical reasons, religious ties, and concerns about the potential reaction of parents.

Despite these challenges, it is noteworthy that almost all participants had some knowledge about HPV, and approximately 60% took their children for vaccination. In this context, Brabin et al. conducted a similar study in schools on parents and guardians to evaluate the acceptance of HPV vaccination. With a response rate of 22%, it was found that most respondents intended to vaccinate their children but feared its potential side effects^13^; interestingly, these data are still consistent with ours, despite a 10-year difference.

Besides, it is already well established that school-based HPV vaccination programs have improved vaccine uptake among adolescents worldwide, and school-based vaccination has proven to be an effective tool in increasing vaccination equity even during the most recent COVID-19 pandemic^14,15^. It has also been shown that after implementing a vaccination promotion program, the HPV vaccination uptake tends to rise, especially in schools where on-site vaccination events take place^16,17^. Thus, it would be reasonable to expect that both private and public schools would greet our effort to further expand the knowledge about HPV and the available vaccination programs. However, there were challenges in establishing contact with institutions from both sectors, and we found it easier to schedule a meeting and deliver questionnaires to private schools in high-income neighborhoods in Santo André. In public schools, the delivery was possible through a meeting with the State Department of Education, which was requested to help contact schools. Nevertheless, return rates were similar between public and private schools, which might be due to the questionnaires being delivered to children in the fourth and fifth grades only, an age group in which parents and guardians tend to accompany children to visits with doctors and the interest in vaccination may be greater.

Furthermore, most participants reported having heard about HPV, with the Internet being the most common source of information. This trend reflects findings from prior studies that were conducted before the implementation of vaccination as a public health policy^18,19^. Although convenient, this method of learning poses risks, as the media can disseminate incorrect information. It must be mentioned, however, that the role of healthcare providers in vaccine uptake cannot be overlooked. More specifically, a study conducted by the Cochrane group has found that positive interactions with frontline healthcare workers were positively associated with vaccination acceptance, thus reinforcing the positive influence that well-trained professionals might have in combating misinformation and providing parents with scientifically accurate guidance^20^.

Still, healthcare providers are not the only influential source of information. In a study on vaccine knowledge among students, mothers, and teachers in public schools in Recife-PE, Silva et al. found that participants pointed to schools as the most effective medium to disseminate knowledge^21^. The discussion about sexuality and the fear of encouraging early sexual practice was one of the obstacles to vaccination education. The fear of adverse reactions to vaccination was also raised.

However, postponing vaccination implies reducing the chances of preventing HPV infections. The lack of knowledge of this information by parents or guardians and the association made between HPV and other STDs may prevent the immunization of children. Thus, the American Society of Clinical Oncology (ASCO) reinforced the need for more aggressive policies to increase the rate of HPV vaccination; the American Cancer Society (ACS), through the National HPV Vaccination Roundtable, created the Vaccinate Adolescents Against Cancer program to promote awareness and education, and in July 2016, the ACS instituted new guidelines, recommending that all men and women receive the vaccine, which shows the importance of vaccination to prevent cancers related to HPV infection^22^.

Recently, the Commission for the Defense of Women's Rights in the Brazilian Chamber of Deputies held a public hearing to expand HPV vaccination, as coverage remains far from the target, especially among boys, whose vaccination rate is below 25%^23^. Our analysis found that living in a low-income neighborhood significantly impacted vaccination rates. Furthermore, currently, we are still facing the aftermath of the COVID-19 pandemic, so interest in the topic discussed in this study has decreased. It has been previously shown that the high HPV vaccination coverage in girls could practically lead to the eradication of cervical cancer in most low- and middle-income countries by the end of the century; however, given the present situation, the HPV infection rate may worsen in the future as the pandemic has caused a major setback in childhood vaccinations worldwide^24,25^.

In summary, addressing HPV in elementary schools is challenging due to logistical and cultural factors, including the stigma associated with discussing sexual health. Reinforcing the relationship between HPV and cancer in public policies is essential, and incorporating scientific discussions on vaccinations in science classes could be a viable strategy not only to educate young people about the importance of the vaccine but also to separate the vaccination campaign from discussions about sexual activity while minimizing the impact of misinformation in the media and on social networks.

## CONCLUSION

Despite being freely available and proven effective, the HPV vaccine remains underutilized. The present study provides insights into the reasons why parents and guardians choose not to vaccinate their children, along with the socioeconomic data for reference. This information may be useful in actions aimed at increasing vaccine coverage in the target population.
